# An Experiment on the Dwell Time Effect of Rubber Seal O-Rings: Friction Force in Intermittent Reciprocating Motion

**DOI:** 10.3390/ma17102427

**Published:** 2024-05-17

**Authors:** Shaoxian Bai, Tao Wang, Jing Yang

**Affiliations:** College of Mechanical Engineering, Zhejiang University of Technology, Hangzhou 310032, China; wt1999801@163.com (T.W.); yangjing@zjut.edu.cn (J.Y.)

**Keywords:** dwell time effect, frictional force, rubber O-ring, intermittent reciprocating motion

## Abstract

The adhesive force between two contact surfaces often leads to an increase in the friction force of the rubber seal O-ring after a certain dwell time, forming dwell time effects and affecting the reliability of sealing. The dwell time effect may result in substantial instability with respect to the frictional behavior of rubber O-rings, which needs to be carefully taken into account in the design of rubber seals. Therefore, in this paper, the dwell time effect of the friction force was studied experimentally for intermittent reciprocating rubber seal O-rings coupled with stainless steel 316L and a sealing air medium. The friction force of three kinds of rubber materials, including fluorine rubber (FPM), silicone rubber (SI), and nitrile rubber (NBR), was measured under different dwell times, compression ratios, and seal pressure. The results showed that there was a rolling frictional force, and the second peak value of the frictional force caused by the O-ring’s rolling under shear action and after the maximum static frictional force was observed at the starting stage of reciprocating motion. For FPM O-rings, the rolling friction force was much greater than the maximum static frictional force at about four times the value of the compression ratio at 9% and seal pressure at 0; moreover, the force was much greater at greater compression ratios. The dwell time effect was significant in the friction forces of rubber O-rings. The friction force increases with an increase in dwell time. The increase in maximum static friction force exceeded 50% after 5 dwell days. The increase in seal pressure led to the disappearance of the rolling friction feature and the rapid increase in friction during the starting stage. Under gas seal pressure conditions, the dwell time effect still led to a significant increase in friction force. The obtained results might provide guidance for the material selection of sealing designs.

## 1. Introduction

A rubber O-ring is a basic part of mechanical seals and magnetic valves, which have been widely applied in many fields, such as petrochemical, nuclear power, and aeronautic equipment [1,2,3,4]. As a common auxiliary sealing element, friction between the rubber O-ring and the corresponding part might affect efficiency, reliability, stick–slip phenomenon, and sealing surface wear in the relative sliding motion [5,6,7,8,9,10]. When a rubber O-ring frequently moves or remains in place for a long time, the friction force on the rubber O-ring might change. The stability of the rubber O-ring’s friction force is an important factor that affects the reliability of sealing [11,12,13,14]. However, the adhesive force between two contact surfaces often leads to an increase in the frictional force of rubber seal O-rings after certain dwell times, forming dwell time effects and affecting the reliability of sealing [15,16,17]. Earlier in 1785, Coulomb [18] first discovered this time effect, noting that friction increased by 140% over a four-day stay. With the demand for modern energy and power equipment for reliable sealing technology and the wide application of rubber O-rings in sealing technology, the problem of the time effect is becoming increasingly prominent, which has become an important factor hindering the reliability design of sealing.

Generally, the time effect in the friction force is believed to be caused by adhesion between two interfaces under dwell times. Davis [19] and Gurney [20] studied the adhesion effect between two interfaces under short residence times. They revealed several unique separation mechanisms between the residence time and the interface’s properties, providing potential ideas for the future analysis of the time effect. Gent [17] studied the bonding strength of an elastic matrix relative to a rigid plate and found that the bonding strength generally increased with an increase in contact time. Based on this analysis, they also found that the residence time had an obvious effect on interface properties, but the relationship between residence time and friction was ignored. Malamut [21] and Kasem [22] presented experimental research on the time effect of static friction forces on spherical polymers, and they found that the time effect of the static friction force is related to the creep of materials; they proposed a theoretical creep model.

For rubber seal O-rings coupled with other materials, the factors affecting friction are more complex. Maegawa [23,24] studied the influence of friction torque on rubber friction and found that the stress distribution on the contact interface had an impact on the level of static friction. Through numerical simulation, it was also found that static friction could be improved by removing it after stopping. Zhou [25] studied the contact evolution of rubber during the sliding process through experiments, and it was confirmed that the contact evolution was affected by the folding and creep of the rubber surface. Our works [26,27,28,29] also found that the friction force was dependent on the rubber material, sealing pressure, compression rate, and other factors, and an obvious maximum value could be observed. However, the above-mentioned study was mainly focused on the changes in static friction force, and it analyzed the influence law of static friction forces from different angles, but the influence of residence time on static friction force was not taken into consideration.

This paper aimed to analyze the dwell time effect in the friction force of long-time intermittent reciprocating rubber seal O-rings. The friction force of different rubber materials coupled with stainless steel 316L was measured for reciprocating rubber O-rings under different compression rates and residence times. The influence of the compression ratio, material, dwell time, and seal pressure on the friction force of rubber O-ring was studied.

## 2. Experiment

### 2.1. Experimental Device and Principle

**Figure 1:**
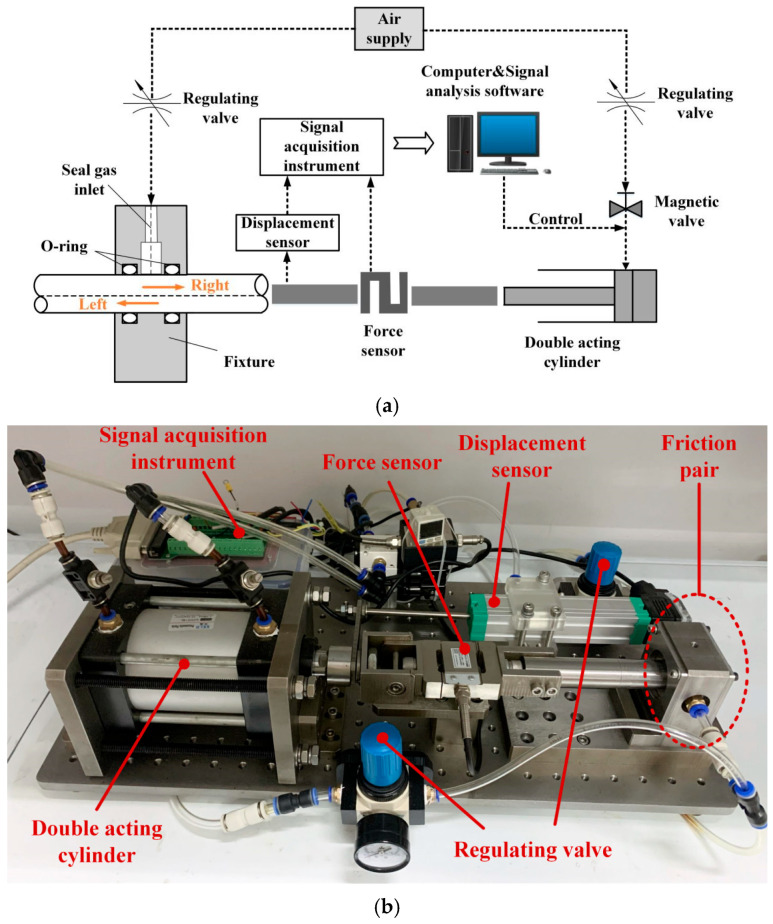
O-ring reciprocating motion test device: (**a**) principal diagram of the experimental device and (**b**) physical experimental device diagram.

Figure 1 shows the schematic diagram of the experimental device for O-ring friction. This device mainly contained two parts, a reciprocating part and a data acquisition part. The reciprocating part included an air source, reciprocating cylinder, force sensor, displacement sensor, and O-ring mounting fixture. The data acquisition section included a data collector and a computer. The measuring accuracy of the displacement sensor used in the experiment was 0.025 mm and that of the force sensor was 0.2 N. The O-ring was in direct contact with the friction rod to form a friction pair. The test bench was fine-tuned to ensure that the reciprocating shaft of the cylinder, the friction rod, and the tension pressure sensor were on a horizontal plane, keeping the reciprocating shaft and the friction rod coaxial.

Since the reciprocating movement of the cylinder was controlled by a computer program, the O-ring’s friction was driven by the reciprocating cylinder to move the friction rod, and a force sensor was used for measurements. The driving force of the cylinder was slowly increased through air pressure until the reciprocating cylinder began to move, and the maximum friction force was obtained using the relationship between the friction force and the displacement image recorded by the computer program. At this time, the friction force was the maximum static friction force required. The O-ring installation fixture had an intake screw port, which can control the inlet pressure. In addition, the compression rate of the O-ring can be controlled by replacing different groove depths and rubber O-rings of different materials and changing dwell times to obtain the friction–displacement relationship of the O-ring under different working conditions.

### 2.2. Samples

**Figure 2:**
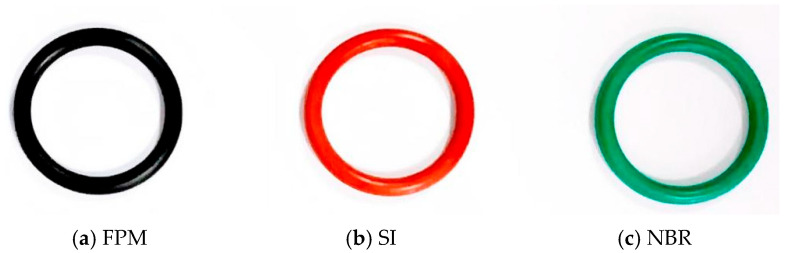
Rubber O-ring samples.

O-rings of 3 rubber materials were tested, including fluorine rubber (FPM), silicone rubber (SI), and nitrile butadiene rubber (NBR), as shown in Figure 2. The section diameter of the rubber O-ring specimen was 3.55 mm, and the inner diameter was 25 mm. Table 1 shows the materials’ characteristics.

**Figure 3:**
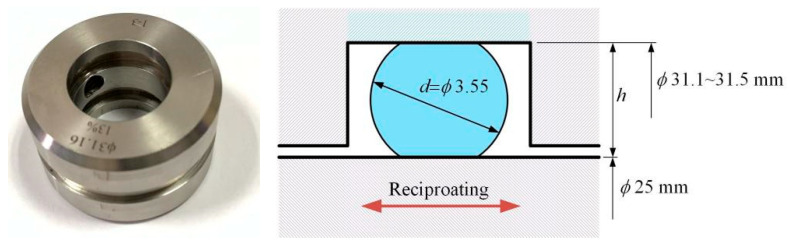
Diagram of O-ring compression installation.

Figure 3 illustrates the compression installation of rubber O-rings in the experiment. Here, the reciprocating rod was made of 2Cr13 stainless steel, and it forms a friction pair with the rubber O-ring. The diameter of the reciprocating rod was 25 mm. Generally, the compression ratio of the rubber O-ring *ε* was calculated using the following formula:

(1) $$\varepsilon =\frac{\left(d-h\right)}{d}\times 100\%$$

where *d* was the section diameter of the O-ring, and *h* was the groove’s depth.

## 3. Results

### 3.1. Reciprocating Friction Characteristics

**Figure 4:**
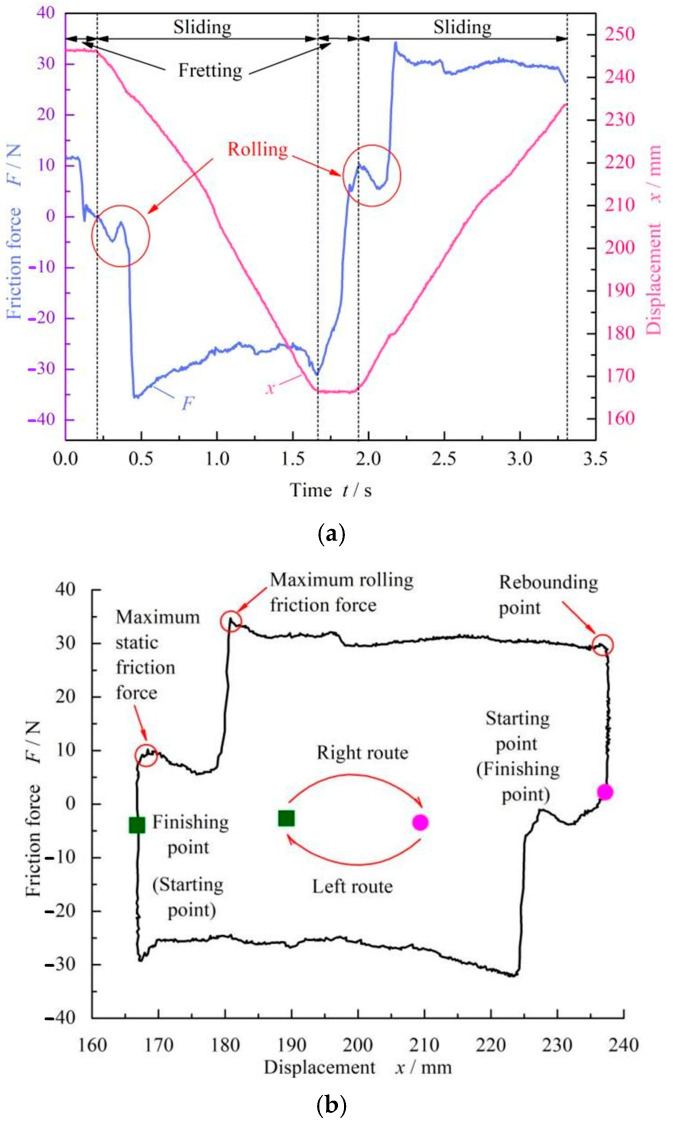
Reciprocating cycle curve of FPM O-rings at *ε* = 9% and *p* = 0 MPa without dwell time: (**a**) friction force and displacement time-varying curves and (**b**) friction force–displacement curve.

Figure 4 shows the cyclic curve of the friction force for the FPM O-ring when the compression rate was 9%. When the cylinder was extended outward, the force sensor was compressed, and the friction force showed a positive value, which was called the right stroke. On the contrary, when the cylinder shrank inward, the force sensor was stretched and showed a negative value, which was called the left stroke.

There were two stages of O-ring motion, fretting and sliding, as shown in Figure 4a. With the extension of the cylinder in the fretting stage, the static friction force increased rapidly, reaching the maximum value. Continually, the O-ring began to slide and the friction force decreased slightly. This phenomenon meant that the O-ring started to roll. After a distance of about 14 mm, the friction force reached a second peak value, which was much greater than the maximum static friction force at about four times the value. Generally, this was caused by the rolling of the O-rings under the shear action of the cylinder slide. Here, this second friction force value was defined as the rolling friction force. Further, Figure 4b gives a curve with respect to friction and displacement, from which it can be seen that the left and right strokes had good correspondence. The right stroke went from exhibiting a rectangular to a circular shape (top half), and the left stroke went from exhibiting a circular to a rectangular shape (bottom half).

It should also be noted that the rubber O-ring will flip when one stroke ends and another begins, so the friction force of the rubber O-ring fluctuates slightly at the connection point of the left and right strokes. A reciprocating pair should have two strokes, and each stroke should have a maximum static friction force. We take the maximum static friction force from the first stroke as the static friction force value in the following analysis. In order to ensure the reliability of the obtained results, the final static friction force was obtained by averaging the value of three measurements.

### 3.2. Dwell Time Effect in Intermittent Reciprocating Motion

**Figure 5:**
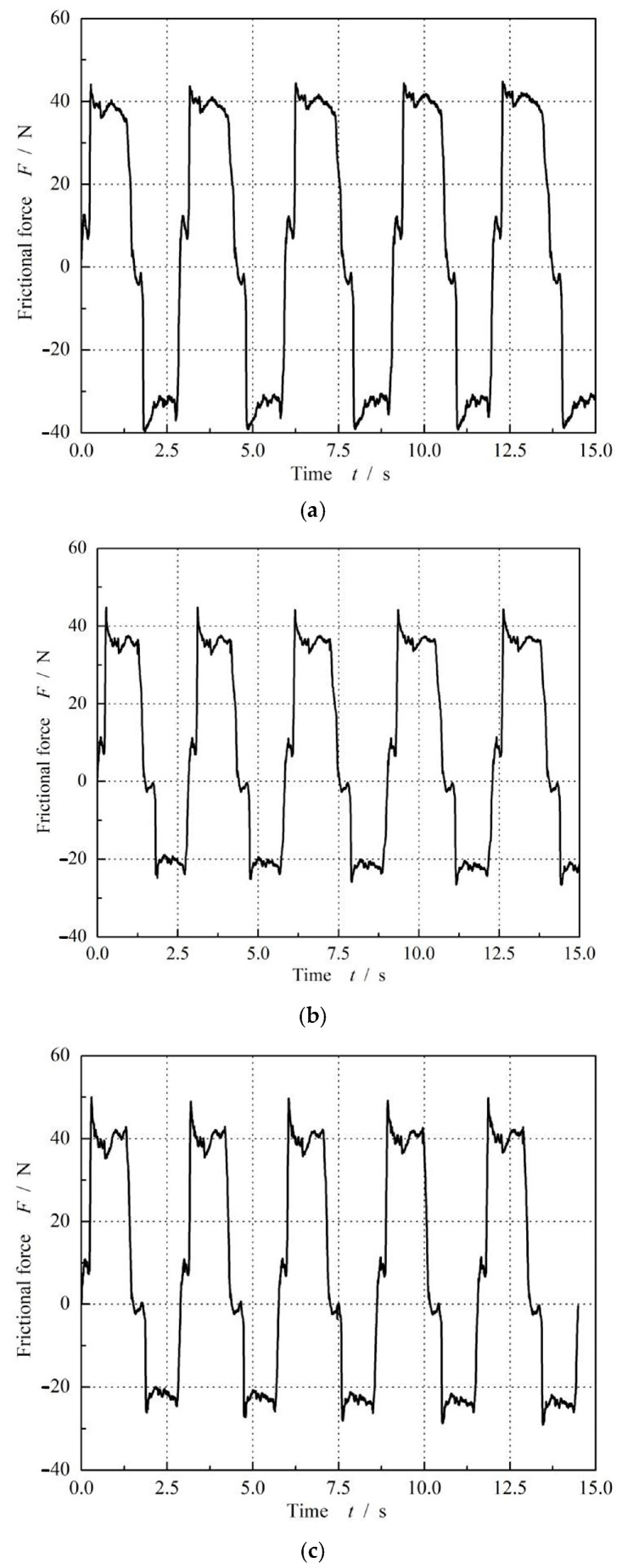

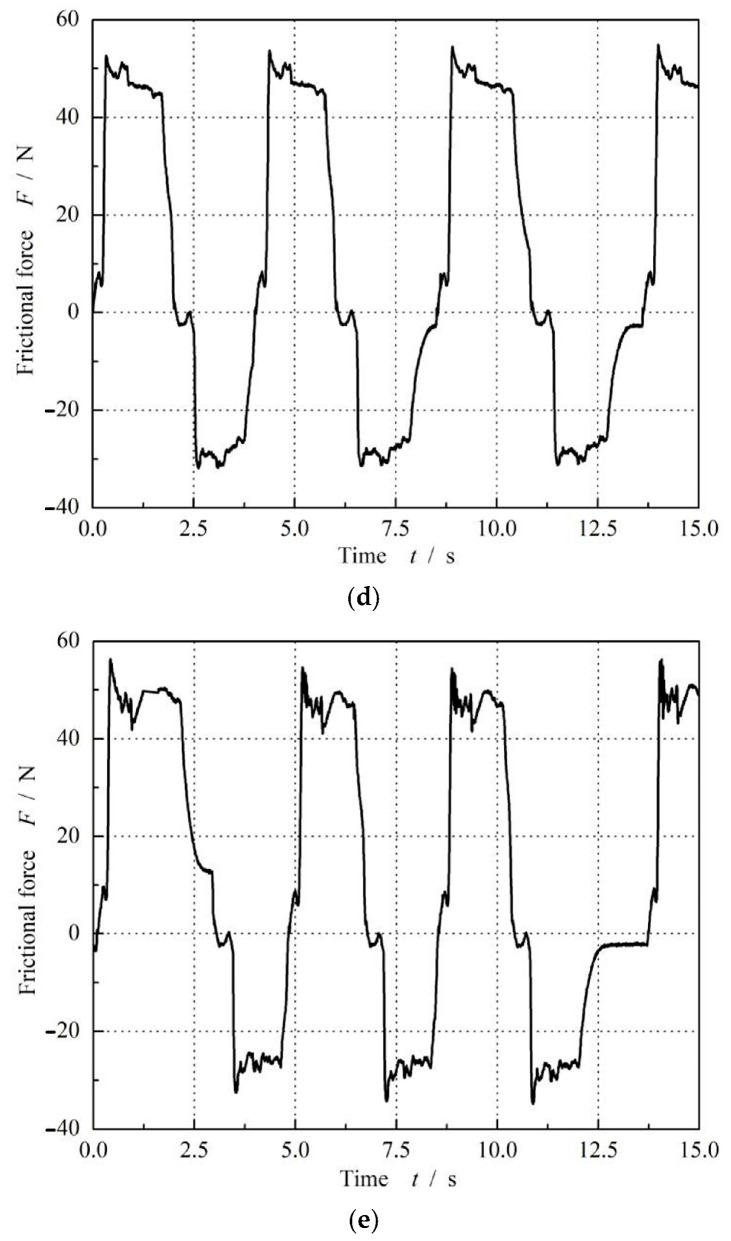
Dwell time effect in the friction force of FPM O-rings at *ε* = 9% and *p* = 0 MPa: (**a**) without dwell. (**b**) One-day dwell. (**c**) Three-day dwell. (**d**) Five-day dwell and (**e**) seven-day dwell.

Figure 5 shows the influence of dwell times on the friction force of FPM O-rings at *ε* = 9% and *p* = 0 MPa. It can be seen that with the increase in dwell time, the friction force shows a significant increase. When the dwell time increased from 1 day to 7 days, the rolling friction force increased from about 45 N to about 57 N. At the same time, it should be noted that the friction force does not change with the number of cycles; that is, the friction force does not decrease as the dwell time increases.

**Figure 6:**
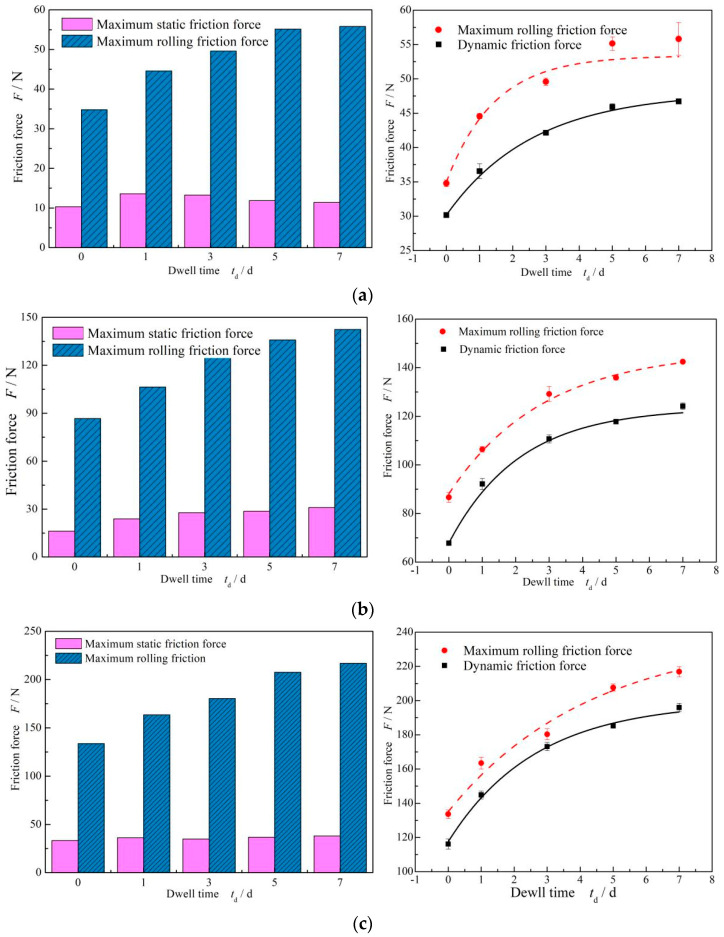
Influence of dwell time on the friction force of FPM O-rings: (**a**) *ε* = 9%; (**b**) *ε* = 12% and (**c**) *ε* = 12%.

Figure 6 shows the influence of the dwell time on the friction force of FPM O-rings at different compression settings. It can be seen that with an increase in residence time, the static friction force and dynamic friction force show an increasing trend, but the growth rate slowed down and became stable after the 5-day dwell period, which had the same characteristics as the theoretical prediction carried out by Kato [30].

In the following section, we further analyzed the influence of dwell time effects on friction under conditions of different O-ring compression rates, sealing pressures, and O-ring materials.

#### 3.2.1. Compression

**Figure 7:**
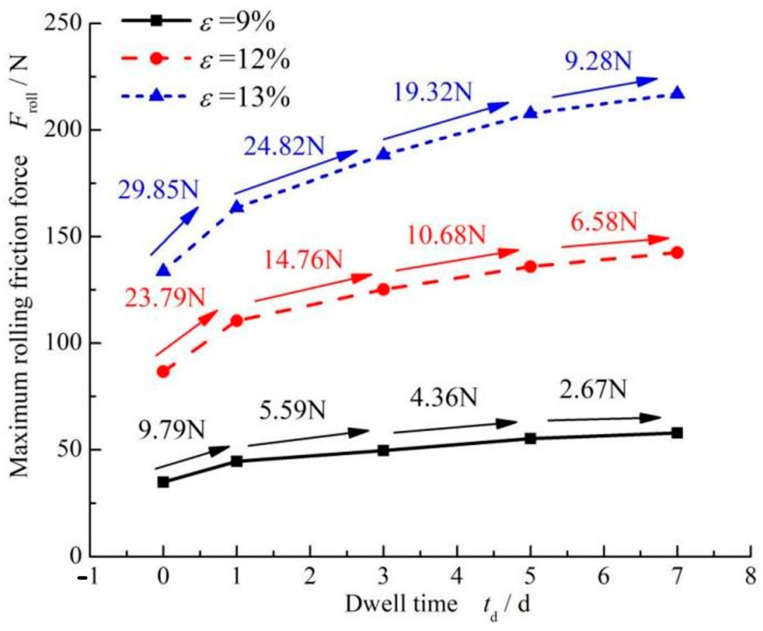
The effect of different compression ratios on the static friction force of FPM O-rings.

Figure 7 shows the variation curve of the maximum static friction force of FPM with respect to dwell time under different compression rates. It can be seen that under the same dwell time, the maximum static friction force increases with the increase in compression ratio. When the compression rate was 9%, the static friction force for one day increased by 9.79 N. When the compression rate increased to 12%, the static friction force increased to 23.79 N when staying for one day, which was almost three times that of 9% compression.

**Figure 8:**
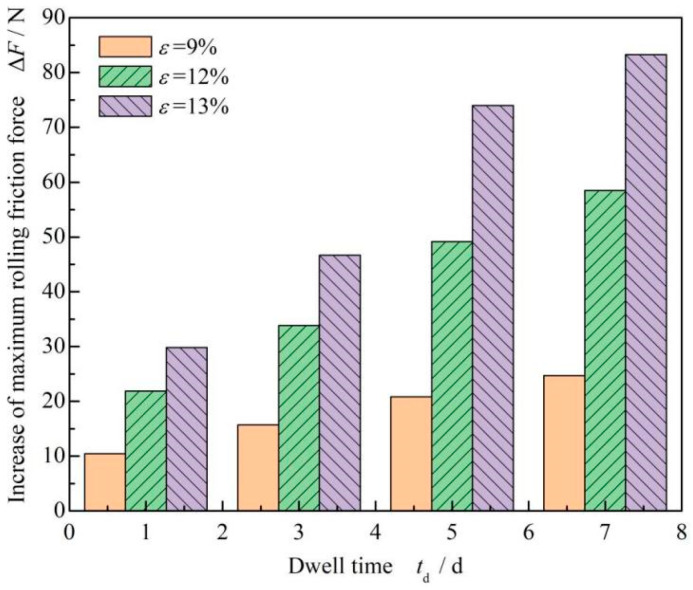
Influence of dwell time on the increase in the maximum static friction force of FPM O-rings under different compression settings.

Figure 8 shows that the change in maximum static friction force increases with dwell times. It can be seen that under three different compression rates, the greater the compression rate, the greater the increase in maximum static friction force. Table 2 shows the increase rate of the maximum static friction force with dwell time. As observed, with the increase in dwell time, the growth rate increases. Moreover, it can also be found that the growth rate of the maximum static friction force after the 5-day dwell period tends to be stable, and the growth rate reached more than 50%. The increase rate after the 1-day dwell period was about 30%, and the increase rate for staying seven days was about 60%. It should be noted that the growth rate of staying static for seven days was doubled compared with that of staying static for one day. Simply put, the increase in the maximum static friction force was proportional to the compression rate of the O-ring, and the growth rate of different compression rates was basically the same.

#### 3.2.2. Seal Pressure

**Figure 9:**
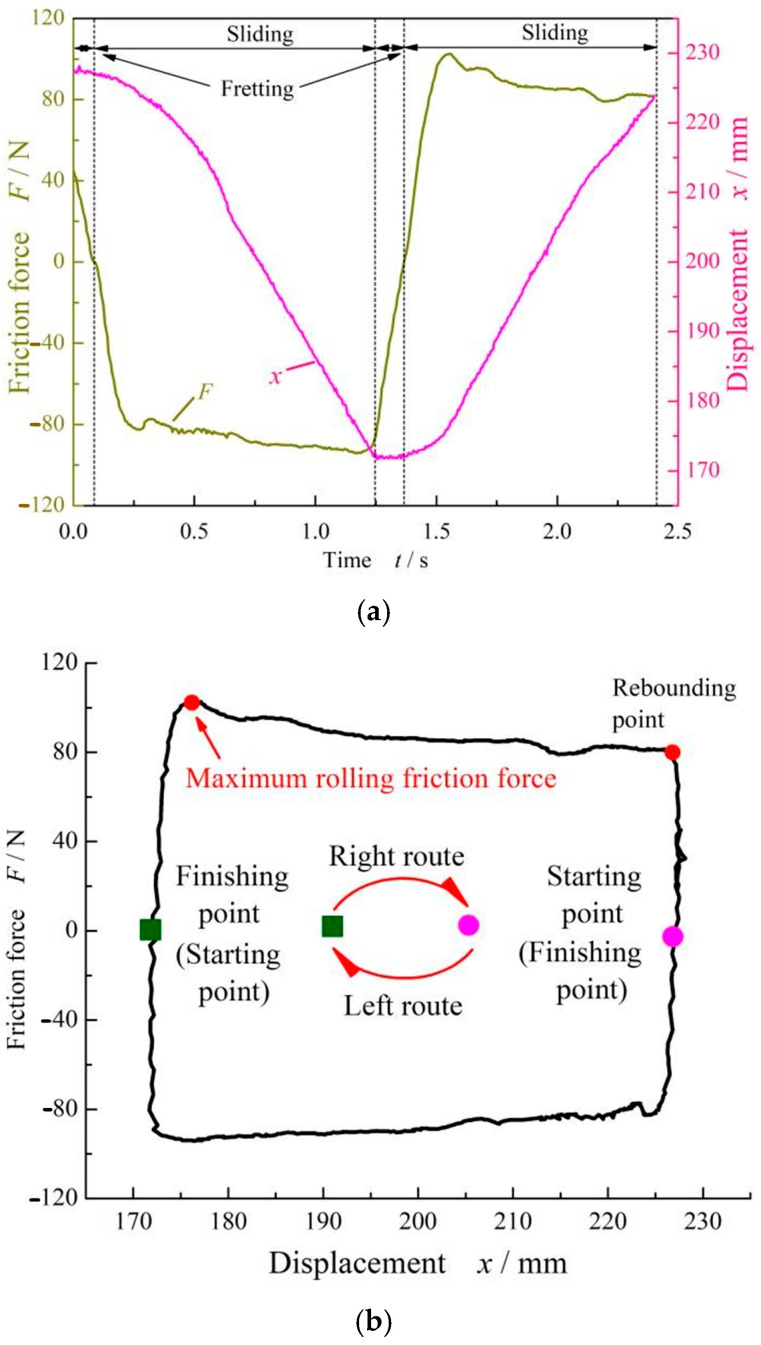
Reciprocating cycle curve of FPM O-rings at *ε* = 9% and *p* = 0.5 MPa without dwell periods: (**a**) friction and displacement time-varying curves. (**b**) Friction–displacement curve.

Figure 9 shows the cyclic curve of the friction force for the FPM O-ring at a compression rate of 9% and seal pressure of 0.5 MPa. Compared to the case of 0 seal pressure, as shown in Figure 3, there was no obvious rolling friction force when the seal pressure was 0.5 MPa. However, the maximum static friction force increased greatly, about 100 N at a seal pressure of 0.5 MPa, which is much higher than the 9.79 N value at 0 seal pressure.

**Figure 10:**
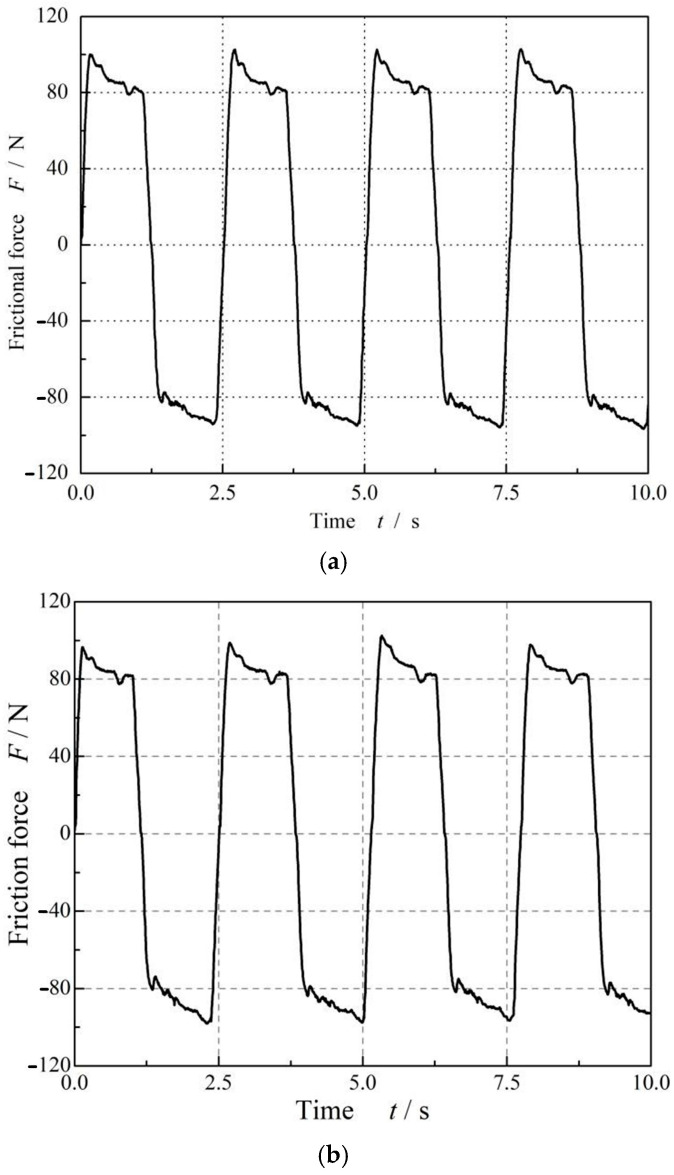
Reciprocating cycle curve of FPM O-rings at *ε* = 9% and *p* = 0.5 MPa: (**a**) without dwell and (**b**) 1-day dwell.

Figure 10 shows the influence of dwell time on the friction force of FPM O-rings at *ε* = 9% and *p* = 0.5 MPa. It can be seen that when the sealing pressure is high, the friction force does not increase significantly with the increase in dwell time. The maximum static friction force is 102.4 N without dwell times, and it is 102.86 N after a 1-day dwell period at 0.5 MPa sealing pressure. Meanwhile, the increase caused by the dwell time remained relatively stable during reciprocating movements.

**Figure 11:**
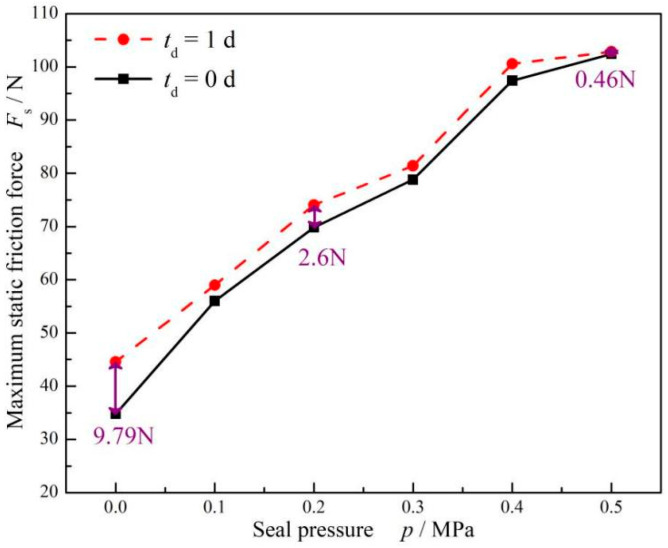
Influence of seal pressure on the maximum static friction force (*ε* = 9%).

Figure 11 shows the influence of seal pressure on the maximum static friction force when the compression rate was 9%. As observed, with the increase in seal pressure, the maximum static friction force presented an increasing trend.

More importantly, the seal pressure may resist the effect of dwell times on the maximum static friction force. When the seal pressure was 0, the dwell time effect caused the maximum static force to increase by about 9.79 N after a one-day dwell period. However, when the seal pressure was kept at 0.2 MPa and 0.5 MPa, the increase in the maximum static friction force decreased to 2.6 N and 0.46 N, respectively, after a one-day dwell period.

#### 3.2.3. O-Ring Material

**Figure 12:**
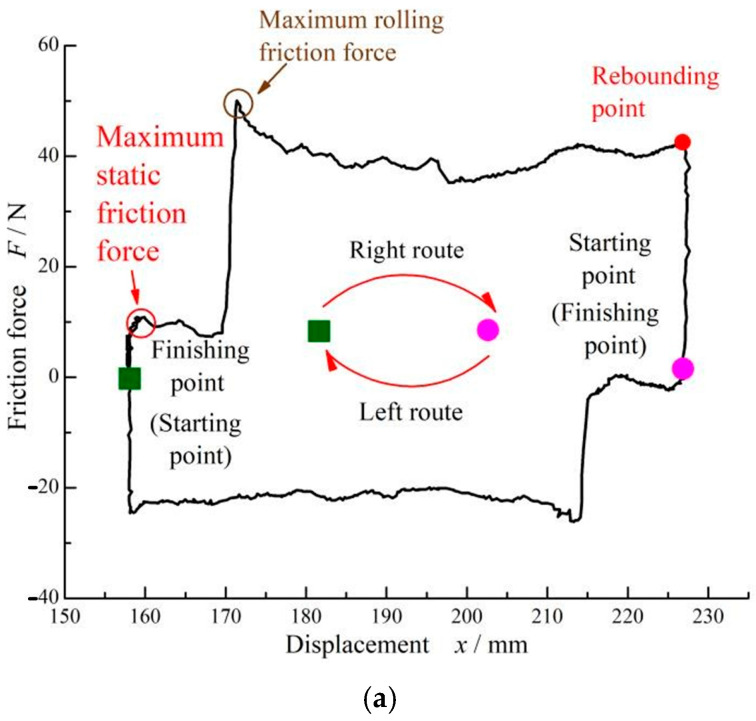

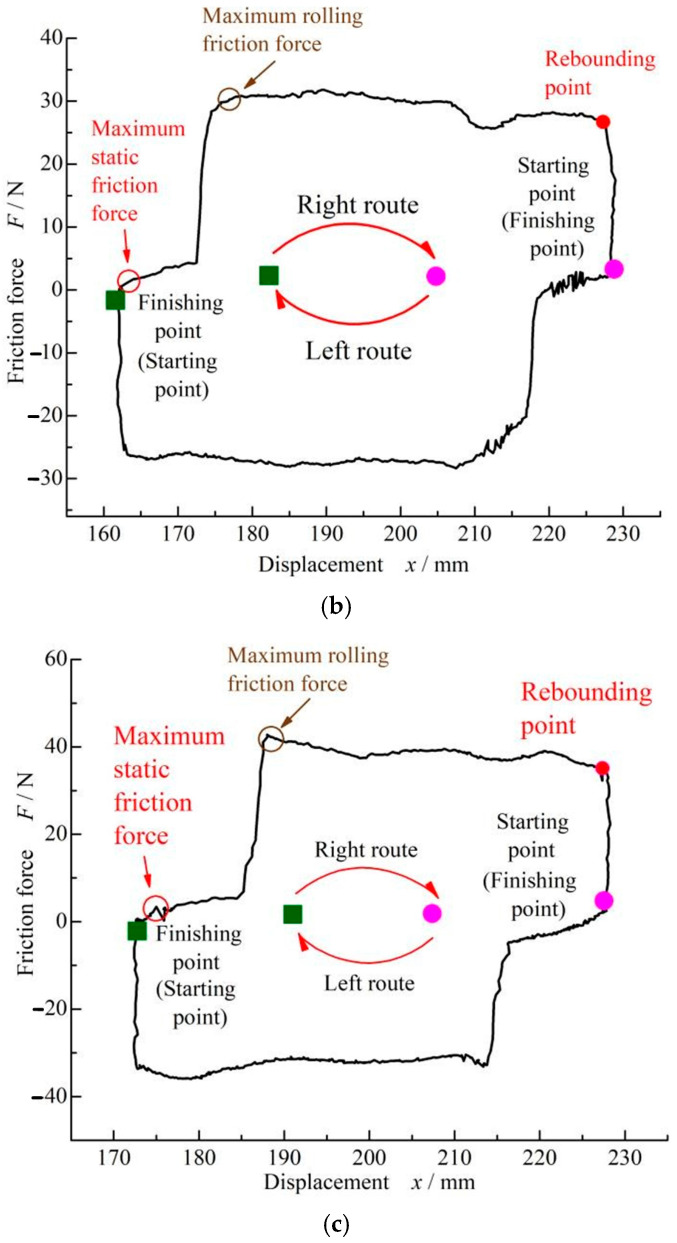
Reciprocating cycle curves of different O-ring material at *ε* = 9% and *p* = 0 after a 3-day dwell period: (**a**) FPM O-ring. (**b**) SI O-ring. (**c**) NBR O-ring.

Figure 12 presents the cyclic curves of the friction force of different O-ring materials at a compression rate of 9% and a seal pressure of 0 MPa after a 3-day dwell period. It can be seen that all three kinds of materials illustrated the same characteristics of friction force. In particular, there was an obvious rolling friction force, resulting in a friction force that was much higher than the maximum static friction force. However, the values of the friction force were different for different materials.

**Figure 13:**
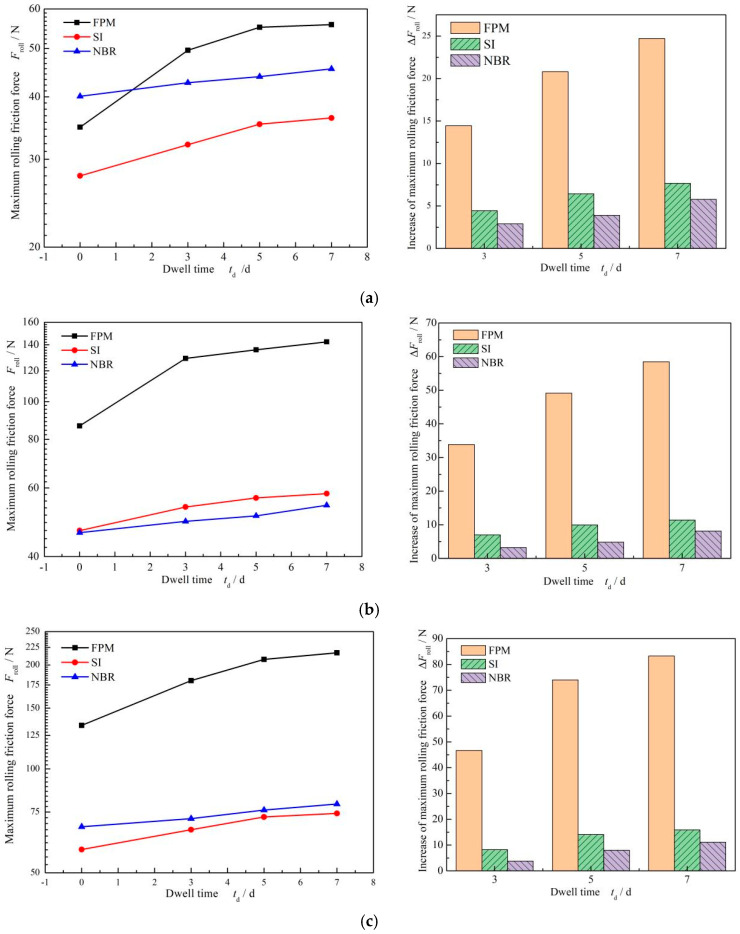
Influence of dwell time on the rolling friction force of different O-ring materials: (**a**) *ε* = 9%. (**b**) *ε* = 12% and (**c**) *ε* = 12%.

Figure 13 shows the influence of dwell times on the maximum rolling friction force of different O-ring materials. With an increase in dwell time, the increase in FPM O-rings was significant, while the increase in SI and NBR was a little different. For the same dwell time, when the compression rate was 9%, the increase in the maximum rolling friction force of fluorine rubber was about three times greater than that of silicone rubber, and when the dwell time was 3 days, the increase in the maximum rolling friction force of the FPM O-ring was about 14.45 N, and the increase in SI was about 4.46 N. When the compression rate increased to 12%, the increase in the maximum rolling friction force of the FPM O-ring was five times larger than two other materials. The maximum rolling friction force of FPM was about 33.84 N, and that of SI was about 6.99 N. So, it can be concluded that the dwell time effect on the maximum rolling friction force of FPM was significant, while the effect on SI and NBR was weak. In addition, the increase in the maximum rolling friction force of FPM was 62% after the 7-day dwell period, while that of SI was 26% and that of NBR was 16%.

## 4. Discussions

For the sealing system, the stability of the friction force of the rubber O-ring is an important factor affecting the reliability of seals. In order to grasp the change rule of the friction force of rubber O-rings, as shown in the above section, we discussed the influence of dwell time, compression ratio, and seal pressure on the friction force.

In the above experiment, we focused on the dwell time effect, which presented an obvious effect on the frictional performance of rubber O-rings. On the whole, the dwell time effect results in the substantial instability of frictional behavior with respect to rubber O-rings. The increase in the maximum static friction force exceeds 50% after a 5-day dwell period, which needs to be taken into account carefully in the design of seals. Meanwhile, the increase in friction force caused by the dwell time was relatively stable in the reciprocating movement. In other words, once the friction force increased after a certain dwell time, it did not decrease. The reason may be that the dwell time effect with respect to friction forces was caused by the time-dependent creep of rubber materials.

In addition, it was also illustrated clearly that, as shown in Figure 11, the seal pressure has an inhibiting effect on the friction force as the dwell time increases. This may be because the increase in seal pressure prevented the O-ring from deforming, resulting in a decrease in the increase in friction force. At different seal pressures, the rubber O-ring had different states of motion. When the seal pressure was 0 MPa, the rubber O-ring rolled with the movement of the piston rod and eventually slid. When the seal pressure was increased to 0.5 MPa, the presence of the seal pressure prevented the O-ring from rolling; therefore, it slid directly.

It should also be noted that the dwell time effect of O-rings with different rubber materials is obviously different. FPM has the fastest friction force growth rate, with a friction force increase rate of more than 60% for a 7-day dwell period. NBR has the slowest friction force growth rate, with a friction force increase rate of less than 20% for a 7-day dwell period, a difference of more than three times. This may be due to the different hardness of different rubber materials, resulting in different deformations of the rubber O-ring. The greater the hardness, the greater the friction force, the more the friction force increases, and the more obvious the time effect of the O-ring’s friction [10].

## 5. Conclusions

This paper experimentally analyzed the dwell time effect on rubber O-ring friction forces during intermittent reciprocating motion. The following conclusions were drawn:

1. There was a rolling frictional force, and the second peak value of the frictional force was caused by the O-ring’s roll under shear velocity and after the maximum static frictional force was observed at the starting stage of reciprocating motion for rubber O-rings. The rolling friction force was much greater than the maximum static frictional force, about 4 times the value in the 9% compression ratio and 0 seal pressure case, which was much greater at higher compression ratios.
2. The dwell time effect was significant in the friction forces of rubber O-rings. The friction force increases with an increase in dwell time. The increase in the maximum static friction force exceeds 50% after the 5-day dwell period. The increase in seal pressure leads to the disappearance of the rolling friction feature, but a rapid increase in friction force occurs during the starting stage. In addition, with the increase in sealing pressure, the time effect of the O-ring’s friction is weakened, and the sealing pressure has an inhibitory effect on the increase in friction.
3. The time effect of the friction force of fluorine rubber is the most obvious, and that of nitrile rubber is the weakest, which may be related to the hardness of the rubber material. The greater the hardness of the material, the greater the friction force; the more the friction force increases, the more obvious the time effect.

## Figures and Tables

**Table 1:** O-ring material characteristics.

Material	Hardness/HA	Temperature Resistance /°C	Tensile Strength /MPa	Elongation/%	Rebound/%
FPM	70–75	−40–200	10–21	100–450	20–40
SI	50–65	−50–180	4–10	90–800	50–85
NBR	65–70	−40–100	6.7–24	400–600	5–65

**Table 2:** Increase in the maximum static friction force of FPM O-rings.

Dwell Time *t*_d_ /d	O-Ring Compression Ratio *ε*/%		
	*ε* = 9%	*ε* = 12%	*ε* = 13%
1	28.14	22.57	26.11
3	42.55	49.12	42.55
5	58.54	57.70	59.94
7	60.47	64.43	62.31

## Data Availability

All data is contained with the article.
